# Effects of Transcutaneous Spinal Direct Current Stimulation on Cognitive and Psychological Outcomes in Multiple Sclerosis: A Preliminary Case Series

**DOI:** 10.3390/biomedicines14051156

**Published:** 2026-05-20

**Authors:** Carmelo Campo, Daniele Saccenti, Angelica De Sandi, Denise Mellace, Simona Mrakic-Sposta, Sara Marceglia, Maurizio Vergari, Andrea Arighi, Alberto Priori, Roberta Ferrucci

**Affiliations:** 1 Department of Business, Law, Economics and Consumer Behaviour “Carlo A. Ricciardi”, IULM University, 20143 Milan, Italy; carmelo.campo2@studenti.iulm.it; 2 Department of Neurosciences and Mental Health, Foundation IRCCS Ca’ Granda Ospedale Maggiore Policlinico, 20122 Milan, Italy; angelica.desandi@policlinico.mi.it (A.D.S.); maurizio.vergari@policlinico.mi.it (M.V.); andrea.arighi@policlinico.mi.it (A.A.); 3 Department of Oncology and Hemato-Oncology, University of Milan, 20122 Milan, Italy; daniele.saccenti@unimi.it (D.S.); denise.mellace@unimi.it (D.M.); 4 Institute of Clinical Physiology, National Research Council (CNR), 20162 Milan, Italy; simona.mrakicsposta@cnr.it; 5 Department of Health Sciences, University of Milan, 20142 Milan, Italy; sara.marceglia@unimi.it (S.M.); alberto.priori@unimi.it (A.P.)

**Keywords:** multiple sclerosis, transcutaneous spinal direct current stimulation, tsDCS, cognitive functioning, depressive symptoms, quality of life

## Abstract

**Introduction**: Multiple Sclerosis (MS) is frequently associated with a range of neurological, cognitive and psychological issues, presenting significant challenges to patients’ Quality of Life (QoL). Among non-invasive neuromodulation techniques, transcutaneous spinal Direct Current Stimulation (tsDCS) is emerging as a potential approach for symptom management in neurological conditions. However, the effects of tsDCS on MS remain poorly explored. Thus, this preliminary study aimed to evaluate the effects of tsDCS on MS symptomatology, focusing on cognitive and psychological variables. **Methods**: Six patients with MS were recruited for a randomized, sham-controlled, double-blind crossover study, and received anodal tsDCS or sham stimulation in two separate sessions at least one month apart. Assessment outcomes included cognitive and attentional-executive functions, depressive symptoms, and several QoL components. The tests were administered at baseline (T0), immediately after treatment (T1), one week (T2) and one month (T3) post-treatment. **Results**: Although protocol-by-time interactions did not reach statistical significance across all measures, protocol-independent improvements over time were observed in various QoL subscales, including Physical Functioning, Role Limitations due to Physical Health, Vitality, Health Distress, and Overall QoL. **Conclusions**: Our findings indicate that tsDCS is a feasible and well-tolerated intervention in patients with MS, with possible implications for QoL. Given the small sample size and the exploratory nature of this study, further research is needed to clarify whether tsDCS may represent a potentially beneficial non-invasive neuromodulation approach for improving well-being in patients with MS across both physical and mental dimensions.

## 1. Introduction

Multiple Sclerosis (MS) is an autoimmune and neurodegenerative disease of the central nervous system characterized by the demyelination of nerve fibers and axonal damage. Despite the progress in MS research and treatment, a critical challenge remains in reducing its significant burden of disease and comorbidity [1,2,3]. The main complications are neurological, including motor, sensory, and autonomic dysfunctions [3,4]. These may manifest as spasticity, pain, or fatigue [3,4], with the latter being significantly linked to disability score [5]. Cognitive deficits are also common in MS [1,2,3,4,5,6,7,8,9]—even when the physical disability has not reached a severe condition [6]—affecting about half of patients [7]. Such impairments are related to cortical and subcortical lesions of the white and gray matter and mainly involve processing speed, executive function, attention, and memory [7,8,9], impacting the ability to perform everyday functional activities [6,7]. Additionally, psychopathological disorders (e.g., depression and anxiety) are prevalent in MS [1,2,3,4,5,10], contributing to a reduction in Quality of Life (QoL) [3,10]. QoL has been suggested as a multifaceted outcome related to a cluster of symptoms [11], and patients with MS frequently experience one of the most severe impacts on QoL compared to those with other chronic illnesses [12]. In this regard, Non-Invasive Brain and Spinal Stimulation (NIBSS) is increasingly used to modulate neural activity and function in pathological conditions, demonstrating efficacy across a wide range of neurological, cognitive, and psychiatric symptoms [11,13,14,15,16,17,18]. Hence—also in light of the limited effectiveness of other existing treatments—NIBSS has been proposed as a promising non-pharmacological treatment for symptom relief in MS [5,11,19,20,21]. In particular, transcranial Direct Current Stimulation (tDCS) in patients with MS has already been shown to improve fatigue [5,19,20,21,22,23], pain [20,21,22], motor function [19,20,21], mood [22], and cognitive deficits [19,21,22]. Some studies have also reported improvements in QoL following tDCS protocols [24,25,26,27]—specifically in activities of daily living, psychological well-being, and coping [24]—while further investigations suggest no changes [28,29,30]. Another non-invasive neuromodulation technique that is attracting interest in this context is transcutaneous spinal Direct Current Stimulation (tsDCS) [16,17]. It applies low-intensity continuous current over the spinal cord to modulate spinal pathway excitability by inducing polarity-dependent changes in neuronal membrane potential [16,17,31]. In particular, anodal tsDCS has demonstrated to increase the excitability of spinal neurons through subthreshold membrane depolarization [31], which could be a polarity-specific effect particularly relevant in MS given the demyelination-induced reduction in spinal circuit transmission [32]. Overall, tsDCS has demonstrated several physical benefits for people with neurological disorders, such as improvements in hypertonia, balance, and tremor [16]. Anodal tsDCS has also shown an inhibitory effect on nociceptive transmission along the spinothalamic tract [33] and a consequent decrease in pain perception [11,34,35], which represents a prevalent and debilitating factor in MS [3,4]. In addition, early evidence indicates that tsDCS could promote mental benefits in psychiatric conditions, such as major depressive disorder [18], and changes in cortical network activity [36,37,38]. These observations suggest an indirect effect even at the supraspinal level, possibly due to signal propagation along ascending afferent pathways [36,37,38,39,40]. However, there is still limited research and application in MS. Only a few studies have investigated the effects of tsDCS among MS patients, reporting positive outcomes for walking function and fatigue [41], neuropathic pain [11,35], and inflammation [42,43]. Consequently, it is essential to explore whether such an intervention may offer other benefits for this condition. In particular, focusing on mental dimensions appears especially relevant, given their substantial impact on patients’ disease burden. Therefore, this preliminary study aimed to evaluate the effects of anodal tsDCS, compared with sham stimulation, on some typically impaired outcomes in MS symptomatology, including cognitive functions, depressive symptoms, and QoL.

## 2. Materials and Methods

### 2.1. Participants

We recruited 6 patients (4 men and 2 women; age range 33–69 years) with an MS diagnosis established according to McDonald’s criteria [44]. Exclusion criteria included relapse or corticosteroid course within the two months preceding the study, pregnancy or breastfeeding, and history of other neurological or psychiatric disorders. During the study period, participants maintained the same medications and dosages used in the previous two months [23]. The study was conducted according to the Declaration of Helsinki and approved by the institutional review board. Patients and their caregivers gave their informed consent before participating.

### 2.2. Experimental Procedure

TsDCS was delivered with an electrical constant direct current stimulator (HDCKit, Newronika, Milan, Italy) connected to two pairs of saline-soaked synthetic sponge surface electrodes. One electrode was positioned over the spinal process of the tenth thoracic vertebra, and the other over the right shoulder. The stimulating electrodes were thick (0.3 cm) and square (35 cm^2^), designed to avoid potentially harmful effects of high current density. We administered two different types of stimulation, anodal and sham (i.e., placebo). For real stimulation, a direct current of 2 mA was delivered for 20 min, once a day for five consecutive days. In the sham condition, electrodes were placed in the same positions, but the stimulator was turned off after 10 s, ensuring participants felt the initial itching sensation without receiving further current [23]. None of the patients experienced adverse effects. We employed a crossover experimental design in which each patient underwent both sham and anodal tsDCS sessions in random order (Sham/Active or Active/Sham sequence), with the two separate experimental sessions held at least one month apart. The patients and the examiner conducting the assessments were blinded to the type of stimulation, while the technician applying tsDCS was aware of its polarity. Assessments were performed by a neuropsychologist of the same foundation at four time points: before treatment (T0, baseline), immediately after the fifth session on day five (T1), one week (T2) and one month (T3) after that final session [15,23,26,27,28,30,34,35,42,43,45].

### 2.3. Measures

The following measures were used in this study. Montreal Cognitive Assessment (MoCA) [46] is a neuropsychological screening test designed to assess global cognitive functioning and detect mild cognitive impairment. Specifically, this test evaluates six different cognitive domains: visuospatial and executive abilities, language, short-term memory, attention, abstract reasoning, and spatiotemporal orientation. The MoCA scoring provides a total score ranging from 0 to 30, with a score of 26 or above typically indicating normal cognitive functioning. In the current study, the valid and reliable Italian version of the MoCA was adopted [47], using its three different forms (available at https://mocacognition.com) randomly assigned across the assessment timepoints to minimize learning effects related to repeated testing [48].

The Symbol Digit Modalities Test (SDMT) [49] is a neuropsychological test that demands information processing speed, working memory and sustained attention, providing an index of executive processing efficiency. It requires participants to match symbols with corresponding digits according to a provided key as quickly and accurately as possible within a time limit of 90 s. In the present study, the test was administered in its standardized written format. Performance was quantified as the total number of correct substitutions completed within the time limit (errors were not counted), with higher scores indicating better performance. The SDMT has been indicated as a valid and reliable measure of cognitive functioning in Multiple Sclerosis [50].

The Posner Cueing Task (PCT) [51] is a neuropsychological test used to assess attentional orienting and shifting, focusing on the ability to allocate and reallocate spatial attention in response to external cues. In the current study, we used an endogenous cue version of the Posner paradigm using a computer-controlled procedure (Wadsworth CogLab software Publishing, Belmont, CA, USA). Participants were first seated at a fixed distance from a computer screen and keyboard and instructed on the task. Before the experimental trials, they completed a short practice block to become familiar with the task instructions. Each trial began with the presentation of a central fixation point followed by a cue, consisting of either a directional arrow indicating the probable location of the upcoming target or a non-informative cue. Subsequently, the target appeared in the cued location (Valid) or in the opposite location (Invalid) or following the non-informative cue (Neutral). Participants were required to indicate as quickly as possible whether the target appeared on the left or right side of the central fixation point by pressing the corresponding keyboard key. Reaction times for correct responses in the Valid and Invalid conditions were recorded and used for analysis. Typically, faster responses following valid cues reflect better attentional orienting, whereas slower responses following invalid cues reflect a difficulty in disengaging attention from the cued location and reorienting it to the target.

Beck Depression Inventory-II (BDI-II) [52] is a self-report questionnaire used to assess the presence and severity of depressive symptoms. It consists of 21 items that can be divided into “somatic-affective” items (e.g., “Tiredness”) and “cognitive” items (e.g., “Past failure”). Each item is rated on a 4-point Likert scale ranging from 0 (symptom absent) to 3 (severe symptom). The total score ranges from 0 to 63, with higher scores indicating a greater depressive symptomatology. Severity of symptoms can be classified as minimal (scores < 13), mild (scores 13–19), moderate (scores 20–28), or severe (scores > 29). The Italian version used in the present study has been confirmed as a valid and reliable measure of depressive symptoms [53].

Short-Form Health Survey-36 (SF-36) [54] is a self-report questionnaire used to assess health-related quality of life and psychophysical well-being in both clinical and healthy populations, measuring various aspects of physical and mental health. It consists of 36 items grouped into eight subscales: Physical Functioning, Role Limitations Due to Emotional Problems, Role Limitations Due to Physical Health, Bodily Pain, Vitality, Social Functioning, Mental Health, and General Health. Items are rated by participants using dichotomous response options and Likert scales ranging from 3 to 6 points. Item responses are converted into a 0–100 standardized score, and the subscale scores are then calculated as the means of the corresponding converted item scores. Each subscale score ranges from 0 (worst health status) to 100 (best health status), with higher scores reflecting a better health condition in the corresponding domain. The Italian version of the SF-36 was used in the current study, which has shown good validity and reliability [55].

Multiple Sclerosis Quality of Life Questionnaire-29 (MSQOL-29) [56] is a self-report questionnaire used to assess both the physical and mental components related to quality of life in the specific context of Multiple Sclerosis. It consists of 29 items organized into eleven subscales: Physical Functioning, Sexual Functioning, Change in Health, Health Perceptions, Bodily Pain, Emotional Well-being, Energy, Cognitive Functioning, Health Distress, Social Functioning, and Overall QoL. Items are rated by participants using Likert scales ranging from 3 to 10 points. Item responses are transformed into a 0–100 standardized score, and the subscale scores are then calculated by averaging the corresponding converted item scores. Subscale scores range from 0 to 100, with higher scores indicating a better health state in the corresponding domain. We used the Italian version of this questionnaire, which has demonstrated good validity, reliability, and factorial structure [57].

### 2.4. Statistical Analysis

Descriptive statistics were used to calculate means and standard deviations for demographics (e.g., age and level of education) and outcome data. Linear mixed-effects (LME) models were fitted to the data to estimate the interaction effect between treatment protocol and assessment time on the several outcomes. This analysis was conducted using the following formula (Wilkinson notation):

*Y* ~ *1* + *Group* * *Time* + *Sequence* + (*1* | *Subject*)

where *Y* represented the outcome measure. Among the fixed-effect factors evaluated, *Group* indicated the stimulation protocol administered to patients with MS (Active vs. Sham), whereas *Time* corresponded to the assessment time points (T0, T1, T2, and T3). The *Group-by-Time* interaction was included to assess whether changes over time differed between the two protocols. A fixed effect of *Sequence* was also included, indicating whether patients underwent active stimulation prior to sham or vice versa. Finally, a random effect of the *Subject* was included. Given the exploratory nature of the study and the limited sample size, the normality of the dependent variables and model residuals was assessed through visual inspection of Q-Q plots and histograms. The LME models were followed by Wald chi-square tests to extract the exact χ^2^ and *p*-values linked to each factor. The p-values were then corrected using the false discovery rate method [58] to control for Type I error. For outcomes reaching statistical significance, post hoc pairwise contrasts between time points were conducted on the estimated marginal means, with p-values adjusted using the Holm method [59]. Statistical significance was determined using a two-tailed p-value threshold of 0.05. The analyses were performed in R (Version 4.5.2; https://www.r-project.org/, accessed on 1 December 2025) [60].

## 3. Results

Descriptive statistics showed that the total sample had a mean age of 53.43 years (SD = 13.14) and a mean level of education of 11.50 years (SD = 4.27). Table 1 summarizes the descriptive statistics (at baseline and across the three timepoints for each protocol) for the subscales of the two QoL measures (MSQOL-29 and SF-36) and the other outcome measures (MoCA, SDMT, PCT conditions, and BDI-II).

Wald chi-square tests applied to LME models showed non-significant interaction effects between group and assessment time on MoCA (χ^2^(3) = 0.09, *p* = 0.994), SDMT (χ^2^(3) = 2.35, *p* = 0.629), PCT-Valid (χ^2^(3) = 0.56, *p* = 0.905), PCT-Invalid (χ^2^(3) = 3.54, *p* = 0.525), and BDI-II (χ^2^(3) = 0.74, *p* = 0.864). The analysis also showed non-significant *Group-by-Time* interaction effects on SF-36 subscales, including Physical Functioning (χ^2^(3) = 1.33, *p* = 0.723), Social Functioning (χ^2^(3) = 2.47, *p* = 0.480), Bodily Pain (χ^2^(3) = 3.63, *p* = 0.470), Role Limitations–Emotional (χ^2^(3) = 1.58, *p* = 0.829), Role Limitations–Physical (χ^2^(3) = 2.03, *p* = 0.707), General Health (χ^2^(3) = 0.48, *p* = 0.923), Mental Health (χ^2^(3) = 1.81, *p* = 0.614), and Vitality (χ^2^(3) = 2.15, *p* = 0.541). Similarly, non-significant *Group-by-Time* interaction effects were shown on MSQOL-29 subscales, including Overall QoL (χ^2^(3) = 3.72, *p* = 0.368), Change in Health (χ^2^(3) = 2.50, *p* = 0.735), Cognitive Functioning (χ^2^(3) = 4.64, *p* = 0.282), Emotional Well-being (χ^2^(3) = 0.55, *p* = 0.908), Energy (χ^2^(3) = 2.26, *p* = 0.651), Health Perceptions (χ^2^(3) = 3.11, *p* = 0.660), Health Distress (χ^2^(3) = 3.64, *p* = 0.303), Bodily Pain (χ^2^(3) = 1.63, *p* = 0.652), Physical Functioning (χ^2^(3) = 1.73, *p* = 0.630), Sexual Functioning (χ^2^(3) = 4.33, *p* = 0.401), and Social Functioning (χ^2^(3) = 1.88, *p* = 0.704).

**Figure 1:**
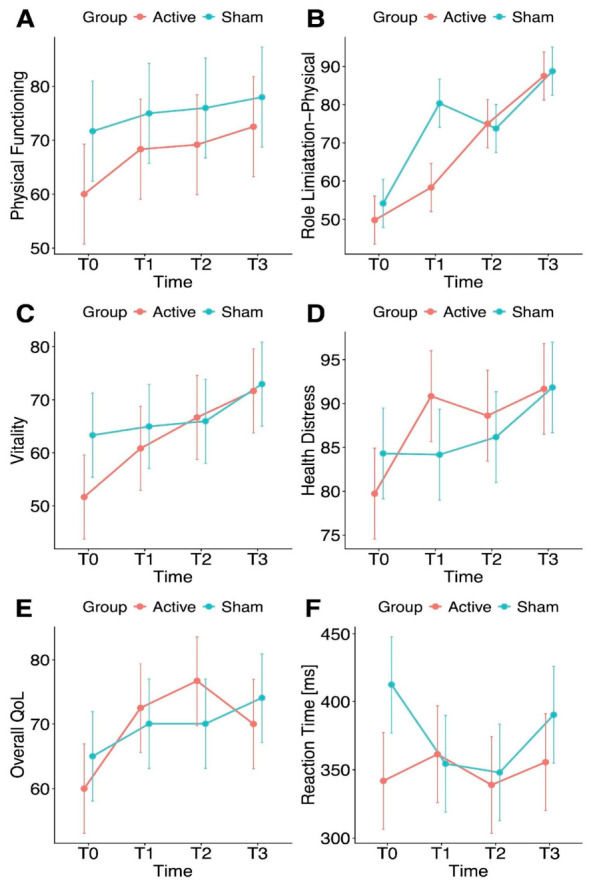
Changes over time in Cognitive and QoL-related domains for the active and sham conditions. Line plots of the fitted data display the estimated marginal means and standard errors for the active and sham conditions across the four time points for outcomes showing significant main effects of Time and/or Group. Regarding the SF-36, significant improvements were observed in patients’ Physical Functioning (**A**), Role Limitations due to Physical Health (**B**), and Vitality (**C**) at T3 compared to baseline. However, a significant main effect of Group was also found for Physical Functioning, with the sham group scoring higher than the active group (**A**). Regarding the MSQOL-29, significant improvements were observed in patients’ Overall QoL at both T2 and T3 compared to baseline (**D**) and in Health Distress at T3 compared to baseline (**E**). A significant main effect of Group was found for the PCT invalid condition (**F**), with the active group showing lower reaction times than the sham group.

**Figure 2:**
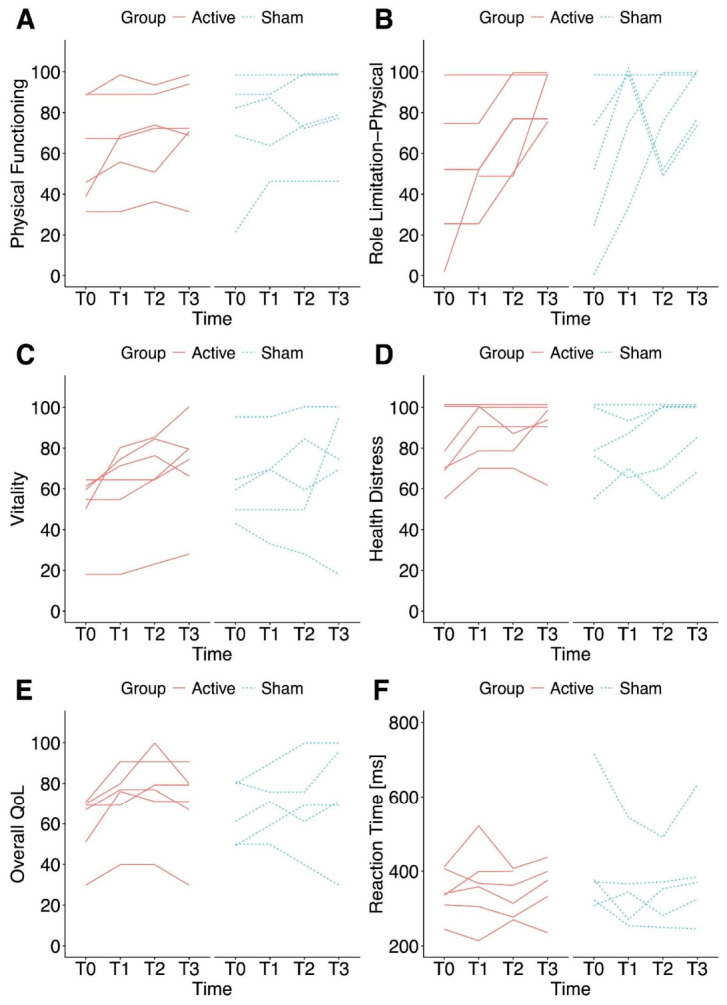
Individual trajectories of Cognitive and QoL-related outcomes for the active and sham conditions. Spaghetti plots of raw data illustrating patients’ individual trajectories across the four time points for outcomes showing significant main effects of Time and/or Group. Regarding the SF-36, a trend toward improvement relative to baseline can be visually appreciated in patients’ Physical Functioning (**A**), Role Limitations Due to Physical Health (**B**), and Vitality (**C**). Turning to the MSQOL-29, a trend toward improvement compared with baseline can be visually observed in patients’ Overall QoL (**D**) and Health Distress (**E**). A minimal difference between conditions can be visually appreciated for the PCT invalid condition (**F**), with the active group tending to exhibit lower reaction times than the sham group, especially at T0 and T3, although this trend was likely influenced by a participant with particularly elevated reaction times at those time points.

Nonetheless, improvements in three SF-36 subscales were observed, irrespective of the stimulation protocol employed. Accordingly, a significant main effect of *Time* was observed when Physical Functioning (χ^2^(3) = 10.39, *p* = 0.026), Role Limitations–Physical (χ^2^(3) = 10.64, *p* = 0.035), and Vitality (χ^2^(3) = 11.33, *p* = 0.025) were set as response variables. Post hoc pairwise comparisons highlighted that the improvements in Physical Functioning (*p* = 0.019), Role Limitations–Physical (*p* = 0.003), and Vitality (*p* = 0.016) were statistically significant at T3 compared to baseline (see Figure 1A–C). Notably, a significant main effect of *Group* was also detected for Physical Functioning (χ^2^(1) = 8.35, *p* = 0.010), indicating that the sham group scored higher than the active group across the four time points. Patients’ individual trajectories are reported in Figure 2A–C. No significant main effects of *Group* or *Time* were observed on the remaining SF-36 domains. No significant main effect of *Sequence* was detected in any case, except when Social Functioning was entered as the dependent variable (χ^2^(1) = 6.96, *p* = 0.021).

Improvements over time were also found in two MSQOL-29 subscales, irrespective of the stimulation protocol. Indeed, a statistically significant main effect of *Time* was observed on both Health Distress (χ^2^(3) = 10.43, *p* = 0.038) and Overall QoL (χ^2^(3) = 13.22, *p* = 0.010). Moreover, a trend toward a statistically significant main effect of *Time* was detected when Cognitive Functioning was set as the dependent measure (χ^2^(3) = 9.66, *p* = 0.054). Post hoc pairwise comparisons showed significant improvements in Health Distress at T3 relative to baseline (*p* = 0.018), whereas significant improvements in Overall QoL were observed at both T2 (*p* = 0.023) and T3 (*p* = 0.049) compared to baseline (see Figure 1D,E). Patients’ individual trajectories are reported in Figure 2D,E. No significant main effects of *Group* or *Time* were observed for the remaining MSQOL-29 domains. No significant main effect of *Sequence* was detected in any case, except when Emotional Well-being was used as the response variable (χ^2^(1) = 18.64, *p* < 0.001).

Turning to the other measures, a significant main effect of *Group* was observed for PCT-Invalid (χ^2^(1) = 5.41, *p* = 0.049), with subjects in the active group exhibiting slower reaction times than those in the sham group, thus indicating greater attentional performance across the four time points (see Figure 1F). Patients’ individual trajectories are reported in Figure 2F. Finally, no statistically significant main effects of *Group*, *Time* or *Sequence* were detected for MoCA, SDMT, PCT-Valid, and BDI-II.

## 4. Discussion

The current preliminary study aimed to evaluate the effects of anodal tsDCS, compared with sham stimulation, on some cognitive and psychological outcomes typically impaired in MS symptomatology. The primary analysis showed the absence of a significant protocol-by-time interaction, indicating that the two treatments did not significantly differ over time across all measures. Nevertheless, significant protocol-independent improvements on specific QoL domains measured by both generic health-related (SF-36) and disease-specific (MSQOL-29) assessment tools were detected across the four timepoints.

Specifically, significant improvements over time were observed in the SF-36 subscales of Role Limitations Due to Physical Health, Physical Functioning, and Vitality, all reaching statistical significance at T3 relative to baseline. These results indicate that patients’ perceived physical health and daily functional abilities increased over the one-month observation period, regardless of the type of stimulation received. Similarly, significant improvements over time were observed in the MSQOL-29 subscales of Health Distress at T3 compared to baseline, and Overall QoL at both T2 and T3 compared to baseline. Again, these results indicate that patients’ perception of disease-related distress and QoL levels improved over the course of the study, without differences between the two protocols. A similar potential improvement was also observed in the MSQOL-29 subscale of Cognitive Functioning. Since these improvements were observed across both the active and sham conditions, they reflect non-specific effects of the intervention that cannot be attributed exclusively to anodal tsDCS. However, even in the absence of both a significant protocol-specific effect and parallel objective measures, increases in these QoL-related self-reported outcomes could still be clinically significant. It represents benefits that patients actually experience in their well-being and everyday functioning, with potential gains across multiple domains of disease burden [11]. A possible explanation for the lack of differences between the two protocols is that both active and sham stimulation may have engaged common mechanisms—such as attention to bodily sensations, therapeutic contact, or expectation of improvement—while anodal tsDCS additionally recruited spinal neuromodulatory pathways [61]. Given the small sample size, this may have produced an overall pattern of improvement that obscured protocol-specific contributions. Moreover, in this crossover design—particularly with the relatively short one-month washout interval and, again, such a limited sample size—cumulative effects across sessions may have limited the detection of differences between the protocols [62]. For example, significant main effects of Sequence emerged for the QoL subscales of Social Functioning and Emotional Well-being, indicating that the order of stimulation influenced patients’ responses on at least some outcomes. The one-month washout interval may not have been sufficient to fully dissipate the effects of anodal tsDCS, which could last several weeks [62]. Thus, it cannot be ruled out that anodal tsDCS may have partially provided a contribution to these improvements that, in the context of this preliminary study, could not be statistically distinguished from sham stimulation. With regard to the observed physical benefits, such a hypothesis would be consistent with previous studies showing benefits of tsDCS on physiological domains (e.g., hypertonia, balance, tremor, and pain) in different neurological disorders [16,34], including MS [11,35,41]. Improvements of this kind would be particularly relevant, as these physical factors often heavily impact the MS-related disability [3,5]. In particular, vitality is a construct strongly linked to fatigue [54], which has been identified as one of the most debilitating symptoms in MS [5]. NIBSS has already been shown to alleviate fatigue in patients with MS [5,19,20,21,22,23], although the only study conducted so far with tsDCS did not report such a benefit [35]. A potential improvement in this subscale would therefore be of considerable clinical relevance. Overall, anodal tsDCS has been shown to modulate spinal excitability, influencing ascending sensory pathways, descending corticospinal pathways, and segmental spinal circuits, thereby producing both motor and sensory effects [31,39,40]. This type of neuromodulation may, in turn, translate into improved and less effortful physical functioning.

Turning to the mental domain, despite having received limited attention in tsDCS research, potential enhancement in this sphere could be particularly interesting given the high prevalence of psychological issues and cognitive deficits in patients with MS as well as their impact on daily functioning and QoL [3,6,7,10]. The absence of a real increment on these variables following tsDCS may be due to the fact that tsDCS primarily modulates spinal cord excitability, unlike conventional tDCS, which directly targets cortical regions implicated in these processes and has been shown to improve cognitive performance [14] and psychological well-being [15,16], even in MS [19,21,22,24]. Accordingly, although it has been suggested that tsDCS could produce indirect effects on supraspinal networks [36,37,38,39,40], it may be more limited in its ability to yield detectable benefits of this kind, particularly on objective measures. Nonetheless, preliminary results appear promising, suggesting that tsDCS could promote mental health [18] and recovery of cognitive functions related to sensorimotor processing [40]. Thus, such impacts may represent secondary effects, mediated by the perception of physical symptom relief and greater ease in performing daily activities. It should also be noted that a significant main effect of Group was detected for the SF-36 subscale of Physical Functioning, with the sham group scoring systematically higher than the active group across all time points. Similarly, a significant main effect of Group was detected for the Invalid condition of PCT, with the active group scoring systematically slower than the sham group across all time points, indicating better cognitive performance. However, in the context of a crossover design, such results may indicate session-level differences in patients’ clinical status—potentially reflecting natural fluctuations in MS symptoms across the two experimental periods, residual carryover from the preceding session, or baseline imbalance—rather than a true differential effect of the stimulation protocols [63,64]. Therefore, they cannot be attributed either to the neuromodulatory effects of anodal tsDCS or to sham stimulation and warrant caution in the interpretation. It is also noteworthy that most improvements emerged at T3. Such a temporal pattern would be consistent with the assumption that non-invasive neuromodulation induces neuroplastic changes that may require time to consolidate into measurable changes, potentially reflecting gradual adaptive mechanisms [65]. However, the absence of a protocol-by-time interaction still precludes any specific attribution to anodal tsDCS. Finally, from a feasibility perspective, tsDCS appeared safe and well-tolerated in our cohort, as no adverse effects were recorded during the study. Given the exploratory nature of this study, it is also worth pointing out that, although the intervention did not fully yield the expected benefits, we did not even observe any evidence of worsening effects on psychological or cognitive outcomes within the study period, which is still a valuable indication of short-term safety and tolerability.

Despite these encouraging results, some limitations need to be acknowledged. The small sample size limits statistical power, the precision of estimates, and generalizability, requiring replication in larger cohorts. The limited sample size did not allow us to investigate potential moderating effects of demographic variables such as sex and age on treatment response, nor to reliably model the period effect, which represents a particularly relevant source of confounding in crossover designs applied to fluctuating conditions such as MS. Moreover, symptom severity, disease duration, and direct measures of motor and sensory symptoms were not specifically accounted for in the present analyses. The relatively short one-month washout interval and the absence of long-term follow-up over one month also restrict our understanding of the intervention’s durability. Accordingly, any significant findings observed in these analyses should be interpreted cautiously, as preliminary and hypothesis-generating signals. Larger and longer future studies should ensure adequate sample diversity and investigate the mechanisms underlying the potential effects—including possible changes in neural connectivity—to provide a more mechanistically grounded interpretation.

## 5. Conclusions

In conclusion, this preliminary study showed that anodal tsDCS did not produce differential effects compared with sham stimulation on psychological and cognitive outcomes in patients with MS. We observed improvements over time in several QoL subscales comparably under both conditions, which therefore cannot be attributed to the active intervention. Nevertheless, these findings highlight the need for further research to determine whether anodal tsDCS—a less extensively investigated protocol compared with cortical stimulation—may represent a feasible, non-pharmacological, and non-invasive approach to improve QoL in patients with MS, across both physical and mental components. If confirmed by further studies, such findings could have important implications for the management of MS, supporting the integration of tsDCS into rehabilitation programs. This approach could offer distinct advantages, such as the direct modulation of sensorimotor pathways underlying MS-related symptoms.

## Figures and Tables

**Table 1:** Descriptive statistics (mean ± standard deviation) at baseline (T0), T1, T2, and T3 for MoCA, SMDT, PCT conditions, BDI-II, and the SF-36 and MSQOL-29 subscales under both active and sham protocols.

Protocol	Variable	T0	T1	T2	T3
Active	MoCA	25.50 ± 2.74	25.83 ± 1.94	24.67 ± 2.34	26.33 ± 1.97
	SDMT	42.67 ± 6.74	47.83 ± 8.68	46.50 ± 7.89	44.50 ± 6.35
	PCT-Valid	371.42 ± 86.40	369.24 ± 120.11	352.06 ± 79.69	352.83 ± 88.42
	PCT-Invalid	341.92 ± 63.40	361.40 ± 103.01	338.94 ± 60.85	356.45 ± 78.98
	BDI-II	4.17 ± 4.36	1.83 ± 1.72	2.50 ± 3.21	2.83 ± 2.71
	SF-36				
	Physical Functioning	60.00 ± 25.88	68.33 ± 25.03	69.17 ± 23.11	72.50 ± 24.85
	Social Functioning	68.67 ± 15.19	79.00 ± 16.98	87.50 ± 20.92	85.17 ± 14.74
	Bodily Pain	89.67 ± 12.42	88.00 ± 13.91	88.67 ± 19.66	95.67 ± 10.61
	Role Limitations–Emotional	83.33 ± 40.82	88.83 ± 27.35	94.33 ± 13.88	88.67 ± 17.56
	Role Limitations–Physical	50.00 ± 39.53	58.33 ± 25.82	75.00 ± 22.36	87.50 ± 13.69
	General Health	49.50 ± 27.54	45.40 ± 23.38	52.17 ± 18.29	55.17 ± 25.86
	Mental Health	74.00 ± 15.95	77.33 ± 17.47	86.00 ± 11.52	79.83 ± 13.45
	Vitality	60.00 ± 25.88	60.83 ± 21.78	66.67 ± 22.29	71.67 ± 23.38
	MSQOL-29				
	Overall QoL	60.00 ± 16.73	72.50 ± 17.25	76.67 ± 20.66	70.00 ± 20.98
	Change in Health	50.00 ± 41.83	66.67 ± 30.28	75.00 ± 31.62	54.17 ± 33.23
	Cognitive Functioning	72.78 ± 13.65	80.56 ± 13.44	80.28 ± 10.77	86.95 ± 9.27
	Emotional Well-being	70.00 ± 10.06	69.72 ± 24.35	75.00 ± 13.58	82.22 ± 13.81
	Energy	56.95 ± 15.29	65.28 ± 28.59	70.83 ± 22.20	69.44 ± 22.77
	Health Perceptions	38.89 ± 25.09	33.33 ± 29.81	44.44 ± 40.37	50.00 ± 27.89
	Health Distress	79.72 ± 17.24	90.83 ± 12.19	88.61 ± 11.37	91.67 ± 14.26
	Bodily Pain	76.95 ± 23.41	78.61 ± 21.87	82.50 ± 27.36	88.61 ± 17.65
	Physical Functioning	65.28 ± 31.37	76.39 ± 22.00	70.83 ± 28.26	73.61 ± 23.22
	Sexual Functioning	5.56 ± 8.61	5.56 ± 10.09	8.33 ± 16.67	6.94 ± 13.35
	Social Functioning	79.17 ± 18.82	79.17 ± 18.82	87.50 ± 20.92	83.33 ± 20.41
Sham	MoCA	26.00 ± 2.10	25.60 ± 2.88	24.80 ± 2.59	26.20 ± 2.17
	SDMT	41.83 ± 8.23	49.20 ± 6.98	47.80 ± 8.56	49.60 ± 10.14
	PCT-Valid	380.97 ± 71.39	349.54 ± 51.83	355.14 ± 86.48	347.65 ± 70.48
	PCT-Invalid	412.35 ± 152.13	355.57 ± 115.82	349.18 ± 94.57	391.42 ± 146.19
	BDI-II	4.83 ± 3.82	3.20 ± 3.96	4.00 ± 5.05	3.20 ± 2.59
	SF-36				
	Physical Functioning	71.67 ± 27.87	77.00 ± 21.97	78.00 ± 34.10	80.00 ± 23.08
	Social Functioning	85.33 ± 16.75	84.80 ± 16.45	85.00 ± 22.36	89.80 ± 10.52
	Bodily Pain	83.67 ± 26.08	88.80 ± 16.35	100.00 ± 0.00	100.00 ± 0.00
	Role Limitations–Emotional	72.17 ± 44.36	66.60 ± 47.20	80.00 ± 44.72	100.00 ± 0.00
	Role Limitations–Physical	54.17 ± 36.80	81.60 ± 29.25	75.00 ± 25.00	90.00 ± 13.69
	General Health	54.00 ± 20.38	57.00 ± 29.38	56.20 ± 23.41	65.00 ± 22.27
	Mental Health	77.33 ± 17.83	78.40 ± 17.34	80.00 ± 16.73	84.00 ± 17.20
	Vitality	63.33 ± 17.51	64.00 ± 22.75	65.00 ± 27.84	72.00 ± 31.74
	MSQOL-29				
	Overall QoL	65.00 ± 13.78	69.00 ± 15.17	69.00 ± 21.91	73.00 ± 27.75
	Change in Health	54.17 ± 18.82	75.00 ± 25.00	60.00 ± 41.83	65.00 ± 33.54
	Cognitive Functioning	75.28 ± 15.14	78.00 ± 12.99	79.67 ± 14.55	76.33 ± 16.52
	Emotional Well-being	73.61 ± 13.23	75.00 ± 17.56	76.33 ± 14.50	80.67 ± 14.41
	Energy	59.72 ± 22.62	66.67 ± 22.82	61.67 ± 29.81	71.67 ± 28.63
	Health Perceptions	33.33 ± 29.81	60.00 ± 27.89	60.00 ± 43.46	53.33 ± 38.01
	Health Distress	84.30 ± 16.85	83.67 ± 14.16	85.67 ± 20.33	91.33 ± 13.25
	Bodily Pain	82.78 ± 19.17	89.00 ± 20.26	89.33 ± 16.77	84.00 ± 21.94
	Physical Functioning	68.06 ± 22.00	78.33 ± 22.52	68.33 ± 32.49	81.67 ± 20.75
	Sexual Functioning	12.50 ± 13.69	10.00 ± 14.91	6.67 ± 9.13	10.00 ± 14.91
	Social Functioning	62.50 ± 34.46	75.00 ± 25.00	75.00 ± 35.36	80.00 ± 20.92

## Data Availability

The data presented in this study are available on request from the corresponding author due to the multicentered nature of the study and related data agreements.

## Abbreviations

The following abbreviations are used in this manuscript:

MS	Multiple Sclerosis
QoL	Quality of Life
NIBSS	Non-Invasive Brain and Spinal Stimulation
tDCS	transcranial Direct Current Stimulation
tsDCS	transcutaneous spinal Direct Current Stimulation
MoCA	Montreal Cognitive Assessment
PCT	Posner Cueing Task
SF-36	Short-Form Health Survey-36
MSQOL-29	Multiple Sclerosis Quality of Life Questionnaire-29
BDI-II	Beck Depression Inventory-II
SDMT	Symbol Digit Modalities Test
LME	Linear Mixed-Effects (model)
